# Racial Differences in Selective Laser Trabeculoplasty Efficacy

**DOI:** 10.5005/jp-journals-10008-1216

**Published:** 2017-01-18

**Authors:** Emil Goosen, Kate Coleman, Linda Visser, William E Sponsel

**Affiliations:** 1 Surgeon, Department of Ophthalmology, Port Elizabeth, Eastern Cape South Africa; 2 Consultant, Department of Ophthalmology, Blackrock Clinic, Dublin, Ireland; 3 Head, Department of Ophthalmology, Nelson Mandela Medical School, Durban, South Africa; 4 Professor, Department of Vision Sciences, University of the Incarnate Word, San Antonio, Texas, USA; Department of Biomedical Engineering, University of Texas, San Antonio, Texas, USA

**Keywords:** Ethnicity, Glaucoma, Intraocular pressure, Selective laser trabeculoplasty.

## Abstract

**Aim:**

Sub-Saharan Africa has a population of 1 billion, with one ophthalmologist per million people. Basic ophthalmic support services are virtually absent for all but a few urban populations. Minimally invasive laser treatment may help. This study reports our initial experience using selective laser trabeculoplasty (SLT) in a mixed-racial population of adult glaucoma patients in Durban, South Africa.

**Study design:**

Institution Review Board approved the 5-year chart review.

**Materials and methods:**

Consecutive glaucomatous adults underwent SLT (Lumenis Selecta) on one or both eyes applying 360° treatment of 120 to 140 closely spaced burns (400 urn spot size for 3 ns; range 1.1-1.4 mJ). Significance of change in intraocuar pressure (IOP) from baseline at 1, 3, 6, and 12 months was assessed by two-tailed paired t-test.

**Results:**

Among 148 eyes of 84 patients (60 African, 21 Indian, 3 Caucasian), 69 had already undergone glaucoma therapy, and 15 untreated *(de novo).* Among all eyes, mean IOP was reduced by >32% with mean IOP < 15 mm Hg from baseline at all four study intervals (p < 0.0001). A 20% reduction in IOP was sustained at 12 months in 90% of African eyes but in only 50% of Indian eyes.

**Conclusion:**

Selective laser trabeculoplasty was effective in producing clinically significant IOP reduction among South African adults with or without prior medical or surgical anti-glaucoma therapy. Socioeconomically comparable individuals of Indian ancestry showed good therapeutic responses, but significantly less efficacious than those observed among Black subjects. Programs to provide first-line SLT management of glaucoma in Africa, where 90% of patients are unable to sustain prescribed medical therapy, appear to be a very appropriate option.

**How to cite this article:**

Goosen E, Coleman K, Visser L, Sponsel WE. Racial Differences in Selective Laser Trabeculoplasty Efficacy. J Curr Glaucoma Pract 2017;11(1):22-27.

## INTRODUCTION

About 285 million people are visually impaired worldwide: 39 million are blind and 246 million have compromising visual impairment (low vision).^1^ Preventable causes account for as nearly 80% of the total global visual impairment burden. About 90% of the world’s visually impaired people live in developing countries.^1-6^ Approximately, 60 million people have glaucomatous optic neuropathy, and an estimated 8.4 million people are blind as a result of glaucoma. By 2020, these figures are expected to increase to 80 million and 11.2 million respectively. Glaucoma is the world’s second leading cause of blindness, and the highest prevalence of open-angle glaucoma occurs in individuals of African ancestry.^7-10^

Primary open-angle glaucoma (POAG) is a disease producing progressive damage to the optic nerve and irreversible functional visual loss that may be partially or fully controlled by daily drops to lower the pressure in the eye. Ethnicity is clearly a major factor in disease susceptibility, with strong epidemiologic evidence that individuals of African and Hispanic descent demonstrate substantially higher prevalence of POAG. Glaucoma is four to seven times more common and also tends to be more severe and less responsive to drug or surgical therapy^11-13^ in individuals of African heritage. Sub-Saharan Africa has one eye specialist per million population, fewer eye clinics, and even fewer affordable sources of antiglaucoma eye drops. The ideal management of POAG would be three pronged, comprising (1) early screening and detection, (2) reliable and predictable treatment options (independent of patient adherence and compliance), and (3) expansion of effective therapies amenable to readily translatable skills development in Africa. In consideration of this set of goals, the authors collaborated with Lumenis (Dr Pazit Pianka) to evaluate the possibility utility of Selecta SLT laser as a means for providing satisfactory intraocular pressure (IOP) control in the African eye. This is part of an ongoing collaboration with the ISGS-sponsored Eyes of Africa coalition of international ophthalmologists adapting cutting edge technology and innovative research in Africa, for Africa.

Durban, once the home of Mohandas Gandhi, has long been known as “the largest Indian city outside of India ... with the highest concentration of Indians overseas.”^14^ Many Indian emigrants to the Durban area became successful landowners after their eventual liberation from indentured servitude following the British Raj, and numerous others followed in their footsteps as free British Commonwealth subjects. Because of strong tradition and imposed government policy, individuals of Indian heritage born in South Africa tended to maintain their strong cultural and genetic identity over the generations since their arrival. Although both the native Africans and those descending ethnically from the Indian subcontinent are both fairly genetically diverse, there are undoubtedly substantive anatomic and genetic ocular characteristics that broadly differentiate the two population subgroups. These are likely to be physiologically subtle and varied, and are beyond the scope of this article.

Laser and surgical treatments have traditionally been the second and third line of therapeutic defense respectively, after topical medications. Prior to the introduction of topical prostaglandin analogs in the 1990s, argon laser trabeculoplasty (ALT) was used frequently to augment the effects of topical medications to help mitigate disease progression. Argon laser trabeculoplasty damages the trabecular meshwork and typically results in only short-term IOP reducing efficacy, with the inability to repeat the treatment. Just as ALT began to take hold as a standard therapeutic option, prostaglandin analogs offered control on monotherapy to a larger proportion of patients, decreasing interest in the early use of trabeculoplasty. In poorly controlled glaucomatous eyes already on maximally tolerated topical therapy, surgery was often chosen as the ethical next step over ALT to avoid unnecessary visual field loss. Unfortunately, among African patients with severe disease fortunate enough to undergo surgery, racially associated tendencies for fibrotic healing of the conjunctiva and Tenon’s capsule often result in bleb compromise and failed pressure control. Antimetabolites that can modulate this tendency are not widely available in Africa, and their use incurs additional risks and challenges. The traditional medicine/laser/surgery sequential treatment hierarchy has been conditionally challenged by the findings of two frequently cited studies. Investigators at Moorfields Hospital in London suggested that surgery first, before medication, might be more efficacious among their predominantly Caucasian patients,^15^ while the Glaucoma Laser Treatment Study (GLT)^16^ suggested that primary argon laser treatment before medication could be advantageous in African Americans.

In 2001, the Federal Drug Administration approved selective laser trabeculoplasty (SLT), a new trabeculoplasty modality that uses pulsed neodymium-doped yttrium aluminum garnet. The pulse duration of only 3 ns is much shorter than the thermal relaxation. Also, unlike ALT (with pulse duration of 100,000,000 ns), no trabecular meshwork scarring occurs with SLT, allowing safely repeatable treatments. Selective laser trabeculoplasty is a laser treatment that reopens obstructed drainage channels in the eye, and one treatment can be reasonably expected to last for 2 years or more. Successful first-line SLT treatment avoids significant compliance and adherence issues, avoids medication-induced toxicity to the delicate conjunctival/ corneal milieu, and very significantly, assuming devices can be made available, potentially avoids major socioeconomic and logistic problems with distribution, quality control, and cost of glaucoma medications.

Selective laser trabeculoplasty technology has now advanced to the point where portable and robust delivery devices could be realistically transported between primary care centers throughout the continent by appropriately skilled personnel. Realini^17^ recently published evidence of remarkable SLT efficacy in St. Lucia, a Caribbean nation with very high glaucoma prevalence among its population of largely West African descent. This study follows on from that landmark paper seeking to gauge the potential utility of SLT therapy in a consecutive clinical cohort of South Africans, comprised largely of two predominant local ethnicities. Our goal was to gauge the potential utility of a more comprehensive SLT therapy campaign in order to provide rapid, minimally invasive, and lasting IOP reduction among the world’s largest, most afflicted, and most underserved glaucoma population.

## MATERIALS AND METHODS

Institutional Review Board approval was obtained for this study from the University of KwaZulu-Natal Biomedical Research Ethics Committee (BREC). Appropriately masked data were entered into a database for more detailed analysis. Informed consent was not required from patients included in this study according to the BREC waiver, as it was a retrospective chart review (http://research.ukzn.ac.za/Research-Ethics/Biomedical-Research-Ethics.aspx) covering all qualifying eyes over a 5-year interval. All the described research at St. Aidan Hospital in Kwazulu, Natal, South Africa adhered to the Declaration of Helsinki. Inclusion criteria required the presence of two or more of the following clinical findings in SLT-lasered eyes with gonioscopically open angles: (1) documented evidence of glaucomatous visual field loss, (2) pathologic disk cup/disk ratio ≥0.6, and (3) IOP elevated ≥24 mm Hg by applanation in at least one eye. Exclusion criteria included the presence of coexisting ocular pathology that could adversely affect visual field, tonometry, gonioscopy, or optic nerve evaluation.

Selective laser trabeculoplasty was performed on either or both eyes of consecutive consenting adult patients using the Selecta laser (Lumenis; Yokneam, Israel). After undergoing pretreatment tonometry by applanation, qualifying glaucomatous eyes immediately received 360° treatment of 120 to 140 closely spaced burns (400 urn spot size for 3 ns). Starting power was 1.1 mJ, adjusted upward if necessary by 0.1 mJ until bubbles were consistently seen (1.1-1.4 mJ). All patients received ketoralac eye drops thrice daily for 28 days post-SLT. Pretreatment with acetazolamide or alpha agonist drops was not performed. Patients were scheduled to return for follow-up and tonometry 1, 3, 6, and 12 months post-SLT. Outcome measures included: (1) Mean change in IOP from the highest level measured within the 4 months prior to the SLT at each time interval, (2) mean change in IOP from the immediate prelaser baseline measurement, (3) proportion of subjects attaining a drop of IOP from baseline of at least 20% (American Academy of Ophthalmology and European Glaucoma Society accepted measure of significance). Subgroup analyses were compiled and compared for patient age, gender, and ethnicity. To distinguish newly diagnosed from established patients receiving prior therapy, two groups were designated: Group I for the *de novo* eyes receiving SLT as their first-line therapy and group II for patients for whom the SLT treatment was added to their existing medication and/or surgery profile. Significance of any change in IOP from baseline at each interval was assessed by two-tailed paired t-test, and age association with efficacy in maintaining IOP control at least 20% below pre-SLT baseline was assessed by linear regression analysis of the bilateral average control value for right and left eyes across the assessment intervals for eyes of patients aged 30 to 39, 40 to 49, 50 to 59, 60 to 69, and 70 to 79 years. The 12-month follow-up data collected after the first laser performed on each eye are those used in this analysis. Eyes undergoing supplemental SLT retreatment at any stage throughout the data accumulation period were documented.

## RESULTS

The study population comprised 148 eyes of 84 patients (41 M, 43F) among whom 60 were Black (B: 71%), 21 Indian (In: 25%), and 3 White (W: 4%). Their ages were normally distributed around a mean age of 58 years (6: 30-39 years, 11: 40-49 years, 26: 50-59 years, 22: 60-69 years, 17: 70-79 years, and 2: 80-89 years). A total of 15 eyes (group I) were previously untreated (6 M, 9 F; 11 B, 4 In, 0 W), and 69 (group II) were under extant therapy (35 M, 34 F; 49 B, 17 In, 3 W). There were no appreciable differences in the use of medications before and at any assessment stage after the SLT, nor any adverse events reported among any of the eyes undergoing laser treatment. There were no differences in the treatment regimen after SLT laser between any of the three ethnic subgroups.

**Graph 1:**
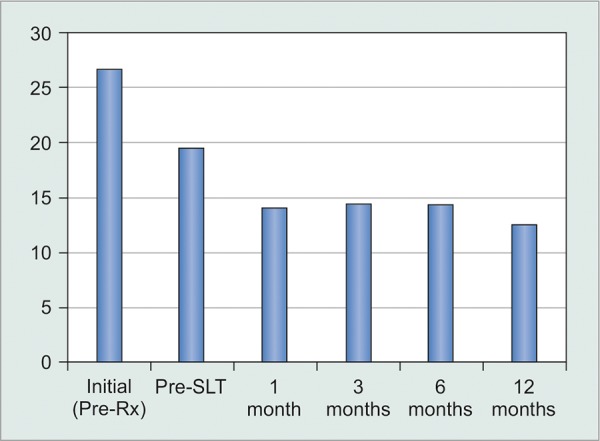
Total group IOP response (p < 0.0001 for pre-SLT *vs* all four post-SLT assessments)

Among the entire study group (n = 148), mean IOP was reduced comparably in both right [oculus dexter (OD)] and left [oculus sinister (OS) eyes from maximal measured pretreatment values at every study interval (Graph 1), from mean values of 27.7 mm Hg OD and 25.9 mm Hg OS and from mean immediate pre-SLT baseline measurements of 19.8 mm Hg OD and 20.1 mm Hg OS. Mean post-SLT IOP values remained below 15 mm Hg at each assessment interval OD and OS. This constituted a reduction of 32% (from 20.0 to 13.6 mm Hg at 12 months; p < 0.0001) from the immediate mean pre-SLT baseline IOP, and of 49% from the highest measured pretreatment IOP value (from 26.8 to 13.6 mm Hg; p < 0.0001).

**Graph 2:**
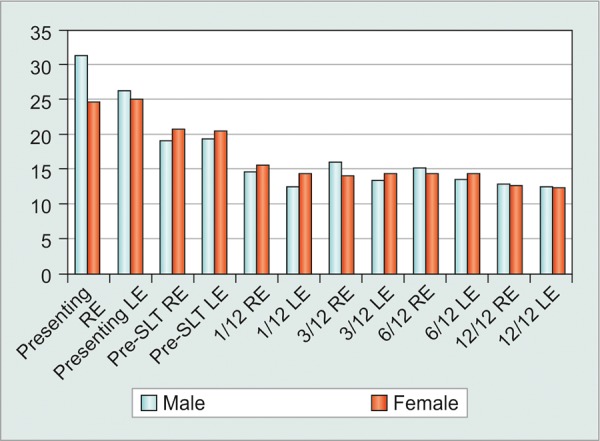
Males dropped IOP by 33.6% at 1 year. Females dropped IOP by 39.18% at 1 year

There was no significant difference in mean IOP between the eyes of male and female patients at any post-SLT interval. Females did, however, have significantly higher mean values for their maximal pretreatment IOP, thereby resulting in a significantly greater mean IOP drop than males relative to that reference value (Graph 2). Moreover, eyes of females required less SLT retreatment (30%) during the 5-year study interval than did those of males (49%).

**Graph 3:**
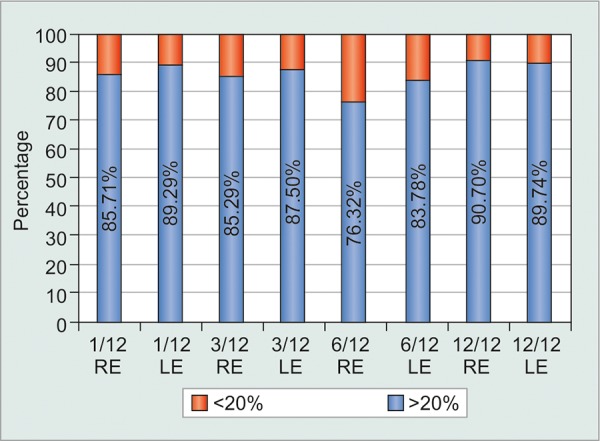
Black patients’ IOP reduction in terms of proportion attaining IOP reduction of >20% (blue) or <20% (red) in each eye at 1, 3, 6, and 12 months (n=68)

**Graph 4:**
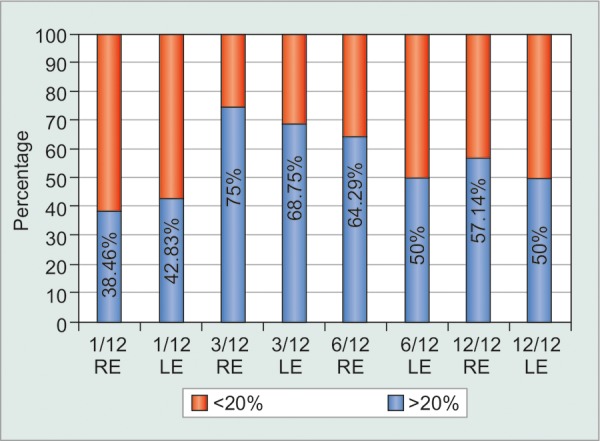
Indian patients’ IOP reduction in terms of proportion attaining IOP reduction of >20% (blue) or <20% (red) in each eye at 1, 3, 6, and 12 months (n=21)

Substantial differences were observed in the mean IOP change from pre-SLT baseline between the eyes of all three racial subgroups, with decreases at 12 months of 42.4% among the eyes of Blacks *vs* 27.8% for eyes of Indian patients, and 28.8% for eyes of Whites. There was accordingly a pronounced difference in proportionate IOP control (20% reduction from pretreatment baseline) between the eyes of Black and Indian subjects (Graphs 3 and 4) with mean bilateral IOP control values between 80 and 90% at each time interval for Blacks *vs* 40 to 70% in Indian eyes. Thus, while the mean IOP reduction among the Black and Indian subgroups was highly significant (p < 0.0001), so was the difference between these subgroups (p < 0.001). The White cohort was too small for meaningful statistical racial comparison.

Patient age demonstrated an association with proportionate IOP control after SLT. Across all the follow-up intervals, maintenance of >20% IOP reduction from baseline ranged from 55 to 71% of eyes in patients aged 30 to 39 years, 63 to 78% of those 40 to 49 years, 62 to 86% of those 50 to 59 years, 68 to 88% of those 60 to 69 years, and 78 to 96% of those 70 to 79 years. The mean IOP reductions from baseline among these groups at the final 1-year post-SLT assessment respectively, were 23, 26, 37, 39, and 45%.

## DISCUSSION

These findings reaffirm the remarkable ocular hypotensive efficacy of SLT among patients of Black African heri-tage.^16^ This response was fully manifest within 1 month of laser administration and sustained throughout the 1-year study interval. This uniform response pattern among Blacks was distinctively different than that seen among similarly treated patients of Indian heritage, who showed a more gradual response pattern. Although response among the eyes of Indian patients was good, it never approached that attained among Blacks.

Our diseased population included many individuals in their 30s or 40s with glaucomatous vision loss. This is consistent with previously published findings among Africans, where “high prevalence, early onset of disease, and an aggressive course combine to produce a high rate of blindness secondary to glaucoma.”^18^ The same article adds, “compounding the problem, there is evidence that blacks are less responsive to both drug and surgical treatment for POAG. Finally, they often have reduced accessibility to treatment and are less aware of the risks of having POAG.” The enhanced relative efficacy of SLT among Blacks stands in stark contrast with the response to incisional surgery, where outcomes among Black patients are generally worse than that arise among Whites. Thus, very fortuitously, a unique racial advantage applies to the most noninvasive, low-risk, and straightforward of all the presently available therapeutic options for glaucoma. Medications are expensive, require long-term compliance, and can be prone to tachyphylaxis and intolerance problems. Incisional surgery is also expensive, with high risk of fibrosis or endophthalmitis, particularly after chronic medication. At present, access to medication, surgery, and SLT laser for glaucoma sufferers throughout Africa is severely limited. There are logistic and financial barriers for all three options, but SLT appears to be the most practicable option available.

The SLT can be used by appropriately qualified practitioners following a short training course, which given the dire shortage of medical staff is critical to success. Furthermore, the treatment could offer a new business model to the African community, allowing job creation, fee income, and cross subsidy of free care for the poorest, as supported by Right to Sight research in eight African countries. The methodology is much less demanding upon administration and follow-up than incisional surgery, and the long-term cost, availability, and sustainability logistics incumbent to topical therapy do not apply to SLT.

In summary, glaucoma prevalence has been confirmed to be as high as 8.5% in studies of Black Africans, in comparison with average prevalence of <2% in the Caucasian eye.^13,19^ It is the second most common cause of needless blindness, affecting as many as 16.5% in one of the few studies available.^18^ Unlike fixable cataract blindness that justifiably occupies nearly all of Sub-Saharan Africa’s few eye surgeons, management of glaucoma is especially critical because lost sight cannot be regained. Training new African cadres and expanding human resources to address glaucoma, to screen for timely quality management, will automatically imply quality eye care services at both remote primary and urban tertiary clinical levels. Once treatment has been carried out, every effort should be made to ensure that appropriate follow-up is maintained. Repeat SLT laser can help to maintain satisfactory IOP control in many patients, and for those in whom this approach is inadequate, surgical intervention may be necessary.

This study was initiated as part of a new concept in eradication of preventable blindness. We believe that cutting-edge technology and innovation can be adapted to reach and prevent blindness for multitudes of previously untreated patients. Focusing attention on a solution for glaucoma in Africa and the genetically predisposed African eye will ultimately advance our understanding of the disease, enhancing treatment and prevention of needless blindness throughout the world. Economic benefits to controlling glaucoma sight loss in the African eye extend to new business models, education and employment opportunities for caregivers, as well as reducing the level of disability in the populations served.

Nongovernmental organizations and politicians have to date been reluctant to implement glaucoma screening programs in Africa, evidently because of the perceived inability to offer effective pressure control, or even long-term supplies of quality medication. Technology is now available to develop and perfect optical coherence tomographic screening, rapid visual field assessments, cloud-based data storage and analysis, and simple IOP assessments. Furthermore, these advances may be offered with minimal training and education. Selective laser trabeculoplasty represents, for the first time, an immediate treatment solution and golden opportunity for intrinsic reward for the detection of glaucoma in African patients. This finally appears to be a glaucoma treatment modality that offers unique advantages among individuals of African descent, rather than severe disadvantages.
